# Evidence for widespread infection of African bats with Crimean-Congo hemorrhagic fever-like viruses

**DOI:** 10.1038/srep26637

**Published:** 2016-05-24

**Authors:** Marcel A. Müller, Stéphanie Devignot, Erik Lattwein, Victor Max Corman, Gaël D. Maganga, Florian Gloza-Rausch, Tabea Binger, Peter Vallo, Petra Emmerich, Veronika M. Cottontail, Marco Tschapka, Samuel Oppong, Jan Felix Drexler, Friedemann Weber, Eric M. Leroy, Christian Drosten

**Affiliations:** 1 University of Bonn Medical Centre, Bonn, Germany; 2 University of Marburg, Institute for Virology, Marburg, Germany; 3 Institute for Virology, FB10-Veterinary Medicine, Justus-Liebig University, Giessen, Germany; 4 EUROIMMUN AG, Lübeck, Germany; 5 German Centre for Infection Research (DZIF)-Partner Site Bonn-Cologne, Bonn, Germany; 6 Centre International de Recherches Médicales de Franceville, Franceville, Gabon; 7 Noctalis, Bad Segeberg, Germany; 8 Institute of Vertebrate Biology, Academy of Sciences of the Czech Republic, Brno, Czech Republic; 9 University of Ulm, Ulm, Germany; 10 Bernhard Nocht Institute for Tropical Medicine, Hamburg, Germany; 11 Smithsonian Tropical Research Institute, Balboa, Panama; 12 Kwame Nkrumah University of Science and Technology, Kumasi, Ghana; 13 Institut de Recherche pour le Développement, Montpellier, France

## Abstract

Crimean Congo hemorrhagic fever virus (CCHFV) is a highly virulent tick-borne pathogen that causes hemorrhagic fever in humans. The geographic range of human CCHF cases largely reflects the presence of ticks. However, highly similar CCHFV lineages occur in geographically distant regions. Tick-infested migratory birds have been suggested, but not confirmed, to contribute to the dispersal. Bats have recently been shown to carry nairoviruses distinct from CCHFV. In order to assess the presence of CCHFV in a wide range of bat species over a wide geographic range, we analyzed 1,135 sera from 16 different bat species collected in Congo, Gabon, Ghana, Germany, and Panama. Using a CCHFV glycoprotein-based indirect immunofluorescence test (IIFT), we identified reactive antibodies in 10.0% (114/1,135) of tested bats, pertaining to 12/16 tested species. Depending on the species, 3.6%–42.9% of cave-dwelling bats and 0.6%–7.1% of foliage-living bats were seropositive (two-tailed t-test, p = 0.0447 cave versus foliage). 11/30 IIFT-reactive sera from 10 different African bat species had neutralizing activity in a virus-like particle assay. Neutralization of full CCHFV was confirmed in 5 of 7 sera. Widespread infection of cave-dwelling bats may indicate a role for bats in the life cycle and geographic dispersal of CCHFV.

Crimean Congo hemorrhagic fever virus (CCHFV; Genus *Nairovirus*, Family *Bunyaviridae*) is a tick-borne pathogen that has caused more than 10,000 documented human infections worldwide, with a case fatality proportion of approximately 10–30%^1^,^2^. Clinical symptoms range from acute febrile illness to severe hemorrhagic fever^1^. To date, the majority of CCHFV cases have been documented in Asia (in particular Russia, Iran, Georgia, Turkey) and Southeast Europe (Greece)^1^,^2^. In Africa, approximately 100 human CCHFV cases have been reported, primarily in Mauritania and South Africa^2^,^3^,^4^. Serological surveys on animals, however, suggest enzootic circulation of CCHFV in different parts of Africa^5^,^6^. The Americas have not reported any autochthonous CCHFV cases^1^,^2^.

Within the genus *Nairovirus* CCHFV represents one of at least seven serogroups. Nairoviruses, classified in serogroups Hughes, Dera Ghazi Khan and Sakhalin, are associated with birds. Viruses in the serogroups Thiafora and Qalyub were identified in shrews and rodents, respectively. Serogroups CCHFV as well as Nairobi sheep disease virus (NSDV) are associated with ungulates^2^,^7^. Only CCHFV, NSDV and the NSDV-related Dugbe virus^1^ are considered human pathogens, while all other nairoviruses are restricted to certain vertebrate host taxa^7^.

CCHFV is known to be transmitted by ticks of the genera *Hyalomma* and *Rhipicephalus*^2^,^6^,^8^. The geographic distribution of CCHFV coincides well with the presence of ticks^9^. However, closely related CCHFV lineages occur in geographically distant regions that do not follow the habitat connectivity of tick hosts. Several studies suggested that tick-infested migratory birds may be responsible for the spatial distribution of CCHFV^9^,^10^,^11^.

Bats (Order: *Chiroptera*) represent the only migratory flying mammals. Bats are commonly infested with soft and hard ticks^12^,^13^, and were recently shown to carry nairoviruses^7^,^14^,^15^. Phylogenetic analyses as well as serological studies based on cross-reactivity in complement fixation tests suggest that all identified bat-associated nairoviruses belong to two novel serogroups that are distantly related to CCHFV^7^. One representative, termed Leopards Hill virus (LPHV), was successfully isolated from samples of the African bat species *Hipposideros gigas* (*H. gigas*). Phenotypic characterization revealed that LPHV causes hemorrhagic disease in mice^15^. Whether LPHV may be pathogenic for other mammals including humans is unknown.

The large diversity of viruses in bats^16^,^17^,^18^,^19^,^20^ together with previous findings of bat nairoviruses^7^,^14^,^15^ encourage a wider and more systematic assessment of the endemicity of nairoviruses in bats. Previous studies were predominantly based on nucleic acid detection and virus isolation, which can only detect viraemic animals. While we do not know the expected frequency of nairovirus viraemia in free-ranging bats, field studies on other RNA viruses with a viraemic infection pattern, including hepadnaviruses^19^, hepaciviruses^17^, filoviruses^21^,^22^,^23^, and bunyaviruses^24^,^25^, indicate very low virus detection rates. Unless very large sample collections are studied, virus detection by PCR lacks the power to reflect the host species range of a given virus, in particular because of a lack of negative predictive value^17^,^18^. Serological techniques are better suited for host range studies because seroprevalence is less sensitive to temporal and spatial variation of infection activity^26^. Serology is also more suitable for the limited sample size that can be achieved in bat-related studies, as most bat species are too small to be bled without destruction.

In the present study we screened 1,135 bat serum samples comprising 16 bat species (migratory and non-migratory) from five different countries for the presence of CCHF-like viruses. The sample was focused on African bat species as nairoviruses have been predominantly detected in the Old World bats^14^,^15^,^16^. We applied a staged set of serological assays including a recombinant glycoprotein (GP)-based indirect immunofluorescence test (IIFT), a novel pseudotype neutralization assay^27^ as well as full virus neutralization tests conducted under biosafety level 4 conditions. Additional molecular screening involved two modified generic nairovirus RT-PCRs based on previously established protocols^28^,^29^,^30^.

## Results

### Bats carry CCHFV GP-reactive antibodies

**Figure 1:**
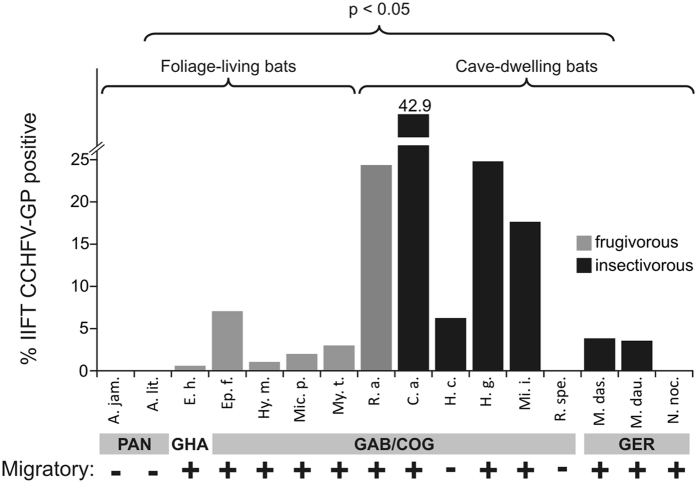
Results of the indirect immunofluorescence test (IIFT) screening for CCHFV antibodies in 1,135 bat serum samples showing highest seropositivity for migratory African cave-dwelling bats. All bat serum samples were analyzed for CCHFV glycoprotein (GP)-reactive antibodies by a modified commercial IIFT (EUROIMMUN). Sera were tested at a dilution of 1:40. Secondary detection was performed with goat-anti bat immunoglobulin G (IgG, 1:1,000) followed by DyLight488–labeled donkey-anti goat Ig (1:100). Gray bars represent frugivorous bat species whereas black bars show insectivorous bat species. A two-tailed t-test was used to compare seropositivity of cave-dwelling versus foliage living bat species (p = 0.0447). Abbreviations: PAN, Panama, GHA, Ghana, GAB, Gabon, COG, Congo, GER, Germany. A. jam. = *Artibeus jamaicensis*, A. lit. = *Artibeus lituratus*, E. h. = *Eidolon helvum*, Ep. f. = *Epomops franqueti*, Hy. m. = *Hypsignathus monstrosus,* Mic. p. = *Micropteropus pusillus*, My. t. = *Myonycteris torquata*, R. a. = *Rousettus aegyptiacus*, C. a. = *Coleura afra*, H. c. = *Hipposideros cf. caffer*, H. g. = *Hipposideros gigas*, Mi. i. = *Miniopterus inflatus*, R. spe. = *Rhinolophus species*, M. das. = *Myotis dasycneme*, M. dau. = *Myotis daubentonii*, N. noc. = *Nyctalus noctula*.

**Figure 2:**
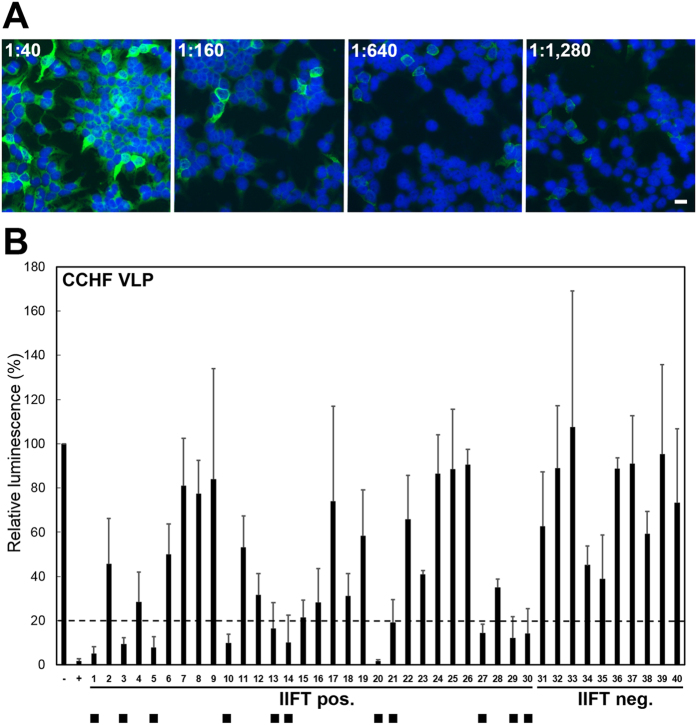
Reactivity of bat serum samples in a recombinant CCHFV GP-based indirect immunofluorescence test (IIFT) and a CCHF virus-like particle (VLP)-based neutralization test (NT). (**A**) Endpoint antibody titration of a CCHFV-reactive bat serum sample by IIFT. A serum sample of *Epomops franqueti* (sample code GB2497) was applied in four different dilutions (range 1:40–1:1,280). Scale bar represents 20 μm. (**B**) Determination of anti-CCHFV neutralizing activity by VLP-based NT. CCHF VLPs were incubated for 1 h at 37 °C with antisera at a final dilution of 1:100. Cells were infected with serum-treated VLPs for 1 h at 37 °C. At 24 h post infection, luciferase activity was measured in cell lysates using the DLA kit. All CCHF VLP-derived *Renilla* values were normalized to an applied transfection control (*Firefly* luciferase values). An untreated CCHF VLP sample (without serum) was used as a negative control (−) and was set to 100% to calculate the relative percentage of luminescence activity. A reduction ≥80% of the normalized *Renilla* luciferase activity (dashed line) was considered as a significant VLP neutralizing activity (positive samples marked by black square). A convalescent mouse-anti CCHFV reference serum (+) was used as a positive control. Mean values and standard deviations were calculated from three independent experiments. Abbreviations: Negative control (−), Positive control (−), numbers 1–30 represent CCHFV IIFT positive bat sera: 1–4 (*Coleura afra*), 5–8 (*Epomops franqueti*), 9 (*Eidolon helvum*), 10–12 (*Hipposideros cf. caffer*), 13–16 (*Hipposideros gigas*), 17 (*Hypsignathus monstrosus*), 18–21 (*Miniopterus inflatus*), 22–26 (*Micropteropus pusillus*), 24–26 (*Myonycteris torquata*), 27–30 (*Rousettus aegyptiacus*). Numbers 31–40 show CCHFV IIFT negative sera: 31 (*Coleura afra*), 32 (*Epomops franqueti*), 33 (*Eidolon helvum*), 34 (*Hipposideros cf. caffer*), 35 (*Hipposideros gigas*), 36 (*Hypsignathus monstrosus*), 37 (*Miniopterus inflatus*), 38 (*Micropteropus pusillus*), 39 (*Myonycteris torquata*), 40 (*Rousettus aegyptiacus*). Detailed information is provided in Supplementary Table 2.

From 2003 through 2011 bats were sampled in Congo, Gabon, Ghana, Germany and Panama. The sample included 1,135 blood or serum specimens from 16 bat species pertaining to six different bat families with variable habitat preference and diet. To assess if bats harbor CCHF-like viruses, we screened bat serum samples for CCHFV GP-reactive antibodies by IIFT. In total, 114 of 1,135 (10.0%) sera from 12 of 16 bat species sampled between 2005 and 2009 in 4/5 countries reacted with recombinant CCHFV GP antigen (range 1.0–57.6%, Table 1, Fig. 1). An example of a reactive bat serum sample is shown in Fig. 2A. IIFT-positive detections were predominantly identified in cave-dwelling, migratory bats including the frugivorous species *Rousettus aegyptiacus* (24.4%; 48/197) as well as the insectivorous species *Coleura afra* (42.9%; 6/14), *Hipposideros cf. caffer* (6.3%; 3/48), *Miniopterus inflatus* (17.6%; 9/51) and *Hipposideros gigas* (24.8%; 32/129) from Congo and Gabon (Fig. 1). CCHFV seropositivity was significantly elevated for bat species that roost in caves versus those that roost in trees (Fig. 1; Supplementary Table 1; two-tailed t-test, p = 0.0447). In particular, bat species from the Batouala cave in Gabon, sampled in 2009, had highest seropositivity (range 18.4–57.6%; Table 1, Supplementary Figure). Age (p = 0.7434), dietary (p = 0.4622), gender (p = 0.4613), migration (p = 0.4788) and seasonality (p = 0.1605) were not associated with differences in seroprevalence (Supplementary Table 1). No antibodies were found in (n = 43) sera from New World bats, corresponding to the notion that CCHFV is an Old World virus^2^.

### Detection of CCHFV neutralizing antibodies in African bat species

**Figure 3:**
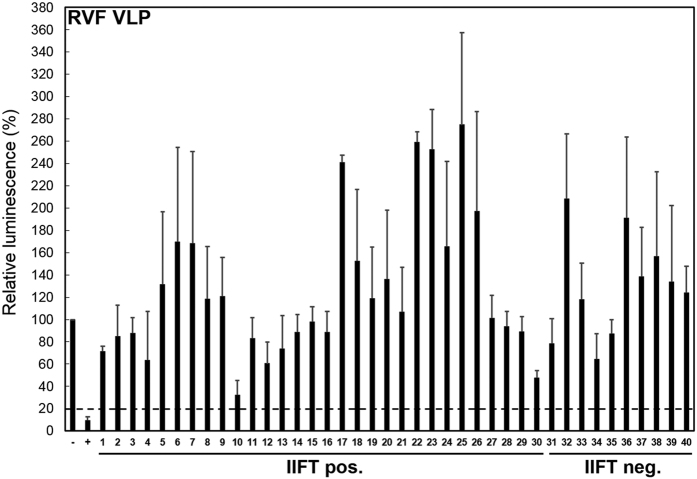
Cross-neutralization control of bat serum samples in a Rift valley fever (RVF) VLP-based neutralization test. To rule out unspecific neutralizing activity of bat serum samples (the same CCHFV IIFT-positive and negative sera as in Fig. 2) RVF VLPs were used with the same serum dilution of 1:100. Incubations and read-outs were performed as described in Fig. 2. A reduction ≥80% of the specific luciferase activity (dashed line) was considered as VLP neutralisation. A convalescent mouse anti-RVFV reference serum was used as positive control (+). Mean values and standard deviations were calculated from three independent experiments. Abbreviations: Negative control (−), Positive control (+), numbers 1–30 represent CCHFV IIFT positive bat sera. Numbers 31–40 show CCHFV IIFT negative sera. For detailed information refer to Figure legend of Fig. 2 and Supplementary Table 2.

As antibody cross-reactivity between CCHFV and viruses from the related NSDV serogroup cannot be completely ruled out by IIFT^31^, specific virus neutralization tests (NT) were done. NT can prove previous infection with CCHFV because there is no cross-neutralization between serogroups^31^. Because NTs require relatively large volumes of serum that cannot be obtained from most bat species, only for 30 of the 114 IIFT-positive sera covering 10/12 bat species, NT assays could be performed. In addition, 10 CCHFV IIFT-negative samples with sufficient volume, representing all 10 assessed bat species, were tested. Sera were tested at a 1:100 dilution in a 96-well format using a recently established CCHF virus-like particle (VLP)-based NT^27^. Out of 30 IIFT-positive sera, 11 showed significant neutralizing activity defined as 80% reduction of luciferase luminescence signal (Fig. 2B, Supplementary Table 2). None of the 10 IIFT-negative control sera had neutralizing activity (Fig. 2B). In parallel, all 40 (30 IIFT-positive, 10 IIFT-negative) bat sera were tested in a Rift Valley fever (RVF) VLP-based NT^32^, all with negative results, ruling out nonspecific neutralization activity in bat sera (Fig. 3, Supplementary Table 2).

For 7 of 11 CCHF VLP-positive sera, enough material was available to conduct additional neutralization tests by a full virus CCHFV neutralization test under biosafety level 4 conditions. Endpoint titration by IIFT in these samples revealed high reciprocal titers between 160 and 1,280 (Table 2, Fig. 2A). Sera were thus titrated in 2-fold serial dilutions in a range of 1:40–1:1,280. The test confirmed full virus neutralizing activity in 5 of 7 sera, with reciprocal titers ranging between 40 (lowest testing dilution due to lack of serum) and 160 (Table 2).

### Lack of evidence for CCHFV-related nucleotide sequences in bat serum samples

To identify CCHFV-related nucleotide sequences in serum samples, we combined three previously established generic RT-PCR protocols that were shown to detect all known nairoviruses^28^,^29^,^30^. Modifications were made to increase the sensitivity for the detection of CCHFV-related nucleotide sequences by applying low annealing temperatures and degenerated oligonucleotides. Viral RNA of CCHFV strain IbAr was used to test oligonucleotides from Lambert *et al*.^29^ in combination with primers from Wölfel and colleagues^30^. The endpoint RT-PCR detection limit of an RT-PCR formulation that used the best combination of primers was between 8 and 80 copies per reaction. An additional RT-PCR assay was developed based on a hemi-nested formulation using primers described in Honig *et al*.^28^ followed by a 2^nd^ round RT-PCR step with novel primers. The sensitivity limit of this assay also ranged between 8 and 80 copies per reaction. Serum samples from *Artibeus jamaicensis* (n = 28), *A. lituratus* (n = 15, both from Panama) as well as *Myotis dasycneme* (n = 26, Germany) were not available for RT-PCR testing due to sample volume limitations (<25 μl). In the remaining 1,067 of 1,135 serologically-tested sera, no CCHFV RNA was found by both RT-PCR formulations. In parallel, these same bat serum sample-derived RNA extracts had successfully been used for the detection of novel paramyxo^18^-, hepe^20^-, hepadna^19^- and flaviviruses^17^, confirming that the material was appropriate for RNA virus testing.

## Discussion

In this assessment of the potential host species range of CCHFV in bats we obtained strong serological evidence for bats constituting a putative host for CCHFV or a closely related virus belonging to the CCHFV serotype. Our failure to directly detect viral RNA may be caused by an overall low infection prevalence that precludes detection in spite of using sensitive RT-PCR assays on a rather large collection of serum samples from bats. We applied two generic pan-nairovirus RT-PCR assays that made use of low stringency amplification conditions to enable the detection of unknown CCHF-related viruses. These conditions led to a maximal 10-fold higher limit of detection (LOD: 8–80 copies/reaction) compared to previously established diagnostic CCHFV real time PCRs with LODs of 5–16 copies per reaction^33^,^34^,^35^,^36^. However, as CCHFV-infected viraemic humans, for example, have viral loads up to 10^9^ viral RNA copies per ml^37^, we assume that viraemic animals would have been identified if present. Nonetheless, we cannot rule out that bats may not experience a pronounced viraemia as observed for birds^38^,^39^.

In addition, sampling time point and/or type of sample may have prevented amplification. In humans it was shown that viremia can be very short (6 days)^37^. Although there is, to our knowledge, no data on nairovirus viraemia in bats, we think that the chances to detect CCHFV RNA in bat samples may be very limited. Previous studies conducted by us and others have already shown that the detection frequency of viral RNA is generally very low (<3%)^17^,^19^.

As shown by Ishii *et al*. the bat-related nairovirus LPHV was primarily detected in lung samples^15^. Therefore, destructive sampling involving the collection of organ tissue of designated bat species may be more promising for nairovirus detection as virus might persist for prolonged periods in parenchymatous organs such as the lung or liver^14^,^15^. In the context of this retrospective study we did not test different organs from bats because most species are protected and were sampled without destruction or samples were exhausted from previous experiments^40^,^41^.

**Figure 4:**
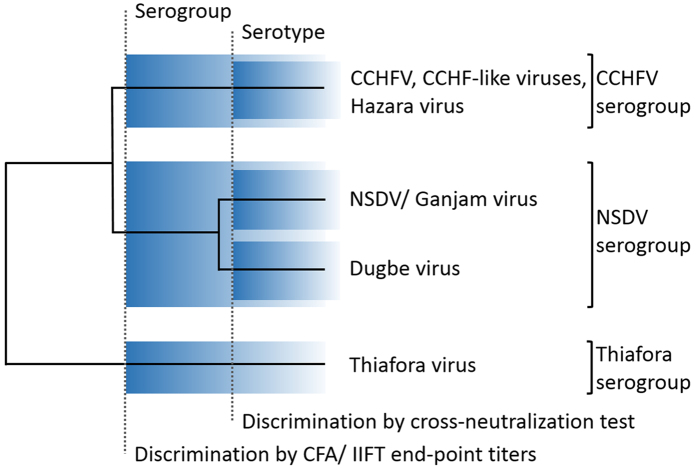
Schematic overview of established serological discrimination criteria for CCHFV and related nairovirus serogroups. The discrimination of nairovirus serogroups and serotypes is based on cross-reactivity patterns in different serological assays^31^,^42^,^43^. Absence of cross-reactivity in complement fixation assay (CFA) and discriminative endpoint titers in indirect immunofluorescence test (IIFT) are used to subdivide serogroups. A differentiation into serotypes can only be achieved by cross-neutralization tests. The detection of high IIFT endpoint titers and neutralizing antibodies in bat serum samples in the present study suggest that bat-associated CCHF-like viruses belong to the CCHFV serogroup.

In spite of this limitation we consider the serological results from our study to be indicative of the presence of members of the CCHFV serogroup in bats. The current demarcation criteria for nairovirus taxa have to rely on the definition of serogroups as long as formal species definitions are not in place. Serogroups based on cross-reactivity in antibody affinity assays reflect phylogeny and can be used to classify novel nairoviruses as they are identified^42^,^43^. For example, complement fixation assays (CFA) and IIFT were applied to discriminate shrew nairoviruses from CCHFV and classify them into a separate serogroup termed Thiafora^44^. Phylogenetically, all members of the Thiafora serogroup form a monophyletic clade in sister relation to a clade containing all members of the CCHFV and NSDV serogroups^7^ (overview shown in Fig. 4). The NSDV serogroup contains NSDV (also named Ganjam virus), and Dugbe virus whose serological classification in one serogroup was again based on cross-reactivity in CFA and IIFT^31^. NT enables further serological discrimination between taxa in serogroups, exemplified by the presence of cross-reactivity but absence of cross-neutralization between Dugbe and NSDV^31^. These taxa might be referred to as two different serotypes within the NSDV serogroup (Fig. 4). The whole NSDV serogroup is discriminated from the CCHFV serogroup, its monophyletic sister taxon, based on absence of cross-reactivity in CFA and decreased IIFT endpoint titers^31^,^43^,^45^. Further sub-differentiation into serotypes based on NT has not been observed within the CCHFV serogroup^42^,^46^. Consequently, the combined reactivity in IIFT and NT suggests that bat species, tested in the present study, carry viruses which are less distinct from CCHFV than Dugbe virus is from NSDV.

Also the height of IIFT titers observed in our study, up to 1:1,280, corresponds to CCHFV-specific titers in experimentally infected wild mammals that reached endpoint titers of 128–1,024 by IIFT^47^. We conclude that African bat species have been infected with CCHFV or a closely related nairovirus in the same serogroup and probably the same species.

The identification of high neutralizing antibody titers (up to 1:160 based on full virus neutralization) indicates that bats experience infections followed by seroconversion, which in case of humans is correlated with clearance of the virus and survival of infection^48^. CCHFV might thus exemplify another highly pathogenic agent that is effectively controlled in bats.

As several cave-dwelling bat species migrate over various distances, bats might contribute to the geographic dispersal of CCHFV in similar ways as hypothesized for birds^9^,^10^,^11^. Future studies should aim to clarify whether the CCHFV strains carried by bats or bat ticks are identical or distinct from strains associated with birds or bird ticks.

Interestingly, we found a predominance of CCHFV antibody-positive bats among cave-dwelling species in particular in the Batouala cave in Gabon. The specific habitat conditions in caves (high population density, host availability, humidity, moderate temperature variations) could potentially enable a virus amplification cycle between bats and ticks^12^,^13^,^49^. Caves harbor many different arthropods including soft and hard ticks. Cave-dwelling animals like ruminants, bird and bats are highly exposed to blood-sucking parasites. Whereas in case of humans and ruminants it is known that CCHFV is predominantly transmitted by hard ticks^2^,^6^,^8^ the role of soft ticks in the CCHFV transmission cycle is less clear. However, soft ticks have been found to carry bunyaviruses^50^ and evidence from CCHFV-endemic countries suggests that certain soft tick species (*Ornithodoros lahorensis, Argas reflexus*) may as well serve as vectors for CCHFV^51^,^52^. Soft ticks are long-lived and blood meals of soft ticks often take only a short period of time (minutes to hours)^53^. Consequently, bats may not be infested by soft ticks during sampling. The low rate of viremia in bats points to a persistence of the virus in ticks rather than bats, encouraging targeted investigations into the pathogen ecology of CCHFV based on cave-associated ticks that can be studied in proximity to bat roosts.

## Methods

### Sampling sites, time points and ethics

In total, N = 1,135 bat serum specimens were collected in five countries (Congo, Gabon, Ghana, Germany, Panama) from 2003 through 2011. A detailed summary of sampling time points is shown in Supplementary Table 3. All animals were handled according to national and European legislation for the protection of animals (EU council directive 86/609/EEC). For all individual sampling sites, study protocols including trapping, sampling and testing of animals were approved by the responsible animal ethics committees as detailed below. Maximum efforts were made to leave animals unharmed or to minimize suffering of animals. All bats were caught with mist nets and blood was taken by vein or heart punctures by trained personnel in accordance with the approved guidelines of the respective authorities. Any surgical procedure was performed under sodium pentobarbital/ketamine anesthesia. Sampling, capturing and sample transport were done as described before^17^,^18^,^19^,^20^ under the following wildlife permits and ethical clearances: Panama (Ethics committee of the Smithsonian Tropical Research Institute (STRI); research permit: STRI2563 (PI VC)- IACUC 100316-1001-18; ethics permit: IACUC 100316-1001-18; export permits: SEX/A-30-11, SEX/A-55-11, SEX/A-81-10, SEX-A-26-10); Ghana (Ministry of Food and Agriculture, Wildlife Division, Forestry Commission, Accra; research permit A04957; ethics permit: CHRPE49/09; export permit: state contract between Ghana and Germany; Congo/Gabon (Ministry of Water and Forest, Libreville; ethics and export permit: 00021/MEFEPA/SG/DGEF/DFC); Germany (Landesamt für Naturschutz und Umwelt (LANUV); ethics permit: LANU 314/5327.74.1.6).

### Indirect immunofluorescence test

CCHFV antibody detection was performed by a modified, commercial, indirect IIFT (EUROIMMUN AG, Lübeck, Germany) according to previous protocols^18^,^54^. EU90 cells (African green monkey kidney) were transfected with plasmids encoding the CCHFV GP (full length GPC precursor of strain IbAr10200). Sera were applied in a 1:40 dilution. Detection involved goat-anti bat IgG (Bethyl, Montgomery, AL, USA) diluted 1:1,000 and Dylight488 labeled donkey-anti goat IgG (1:100). Analysis was performed with a Motic Immunofluorescence microscope (Zeiss, Jena, Germany).

### Neutralization test using CCHFV virus-like particles

Biosafe NT were done using CCHF VLP consisting of CCHFV structural proteins and a *Renilla* luciferase minigenome unable to produce infectious viral progeny^27^. In parallel, a pGL3-*Firefly* construct was applied as transfection control. VLPs were incubated for 1 h at 37 °C with antisera at a final dilution of 1:100. Cells were infected with serum-treated VLPs for 1 h at 37 °C. At 24 h post infection, luciferase activities were measured in cell lysates using the Dual-Luciferase Reporter Assay System (Promega, Mannheim, Germany). The same VLP methodology was described earlier for Rift Valley Fever virus (RVFV) and was used in parallel as a control antigen in this study^32^.

### Neutralization test by CCHFV strain IbAr

Sera reactive against CCHF VLP at dilution 1:100 were confirmed by quantitative CCHFV NT in a biosafety level 4 facility. Serial two-fold dilutions (from 1:40–1:1,280) of serum were incubated with ~1,000 focus-forming units (FFU) of CCHFV (IbAr strain, originally isolated from a Nigerian camel tick^5^) for 1 h at 37 °C. Vero E6 cells were infected in triplicate with serum-treated CCHFV for 1 h at 37 °C. After 20 h cells were fixed and stained with rabbit polyclonal antibody raised against the CCHFV nucleoprotein and detected by Alexa Fluor 488 donkey anti rabbit conjugate (Thermo Scientific, Braunschweig, Germany).

### Viral nucleic acid detection

For nucleic acid detection viral RNA was extracted with the QIAamp viral RNA mini kit (QIAGEN, Hilden, Germany). A combination of three previously established generic nairovirus PCR protocols were applied with modifications^28^,^29^,^30^. Oligonucleotides from Lambert *et al*.^29^ were used in multiple combinations with primers described by Wölfel and colleagues^30^. Tenfold dilution series of viral RNA of CCHFV strain IbAr were used to determine the sensitivity of the PCR assays. Optimal amplification of CCHFV RNA was identified using the oligonucleotide combinations Nairo-F (*ATG ATT GC5 AAY AG5 AAY TTY AA*) and Nairo-R (*ACA GCA RTG 5AT 5GG 5CC CCA YTT,* 1^st^ round) and cc1a-c-F (*GTG CCA CTG ATG ATG CAC AAA AGG ATT CCA TCT*) and Nairo-R (2^nd^ round) in a hemi-nested PCR protocol. The use of the nucleoside inosine is indicated by the number 5. In a second PCR assay, which was based on the Honig *et al*.^28^ protocol, the published 1^st^ round of PCR amplification by oligonucleotides Nairo-F and Nairo-R was followed by a newly introduced 2^nd^ round PCR using oligonucleotides Nairo-Fnest (*CCA AGA AGY GT5 AGR AGY AAR GT*) and Nairo-Rnest (*TTG GGC CCC ACT T5G TRT TRT C5C C*). The PCR protocol was as follows: 1^st^ round PCR including reverse transcription 50 °C, 20 min, 94 °C 3 min, 10× (94 °C 15 s; annealing for 20 s at decreasing temperature starting at 60 °C to 50 °C; 72 °C 35 s), 40× (94 °C 15 s, 50 °C 20 s, 72 °C, 35 s). 2^nd^ round PCR same condition as the 1^st^ round without reverse transcription.

### Statistics

A two-tailed t-test with equal variance (www.openepi.com) was used to correlate CCHFV seroprevalence in different bat species with designated features (age: adult/subadult versus juvenile; dietary: frugivorous versus insectivorous; gender: male versus female; migration: migratory versus resident; roosting: foliage versus cave-living; seasonality: wet versus dry). For all calculations only bat species with a positive CCHFV-IIFT result were included.

## Additional Information

**How to cite this article**: Müller, M. A. *et al*. Evidence for widespread infection of African bats with Crimean-Congo hemorrhagic fever-like viruses. *Sci. Rep.* **6**, 26637; doi: 10.1038/srep26637 (2016).

## Supplementary Material

Supplementary Information

## Figures and Tables

**Table 1:** Indirect immunofluorescence test (IIFT), CCHF virus-like particle and CCHFV-based neutralization test for CCHFV antibodies on bat serum samples from five countries accumulated in the years 2003 to 2011.

Bat species	Country	Site	Year	Diet	Migratory*	IIFT CCHFV-GP	CCHFV VLP assay	CCHFV NT
						n positive/tested (percentage)		
**Foliage-living bats**								
*Artibeus jamaicensis*	Panama	BCI	2011	F	N	0/28 (0.0)	ND	ND
*Artibeus lituratus*	Panama	BCI	2011	F	N	0/15 (0.0)	ND	ND
*Eidolon helvum*	Ghana	Kumasi Zoo	2009	F	Y	1/96 (1.0)	0/1 (0.0)	ND
			2010			0/71 (0.0)	ND	ND
*Epomops franqueti*	Gabon	Lepaka	2008	F	Y	2/44 (4.5)	0/2 (0.0)	ND
	Congo	Lebango	2006			5/26 (19.2)	1/2 (50.0)	0/1 (0.0)
		Mbomo	2006			0/29 (0.0)	ND	ND
*Hypsignathus monstrosus*	Gabon	Cimetière	2005	F	Y	0/1 (0.0)	ND	ND
		Ekata	2006			0/7 (0.0)	ND	ND
		Hopital	2005			0/1 (0.0)	ND	ND
		Lepaka	2008			0/13 (0.0)	ND	ND
	Congo	Lebango	2005			0/18 (0.0)	ND	ND
			2006			0/19 (0.0)	ND	ND
		Mbomo	2003			0/17 (0.0)	ND	ND
			2005			0/17 (0.0)	ND	ND
			2006			1/8 (12.5)	0/1 (0.0)	ND
*Micropteropus pusillus*	Gabon	Cimetière	2005	F	Y	1/4 (25.0)	0/1 (0.0)	ND
		Hopital	2005			0/4 (0.0)	ND	ND
		Hopital S	2005			0/4 (0.0)	ND	ND
		Keri	2005			0/5 (0.0)	ND	ND
		Ngounie	2005			0/1 (0.0)	ND	ND
	Congo	Lebango	2005			0/1 (0.0)	ND	ND
			2006			0/2 (0.0)	ND	ND
		Mbomo	2005			0/12 (0.0)	ND	ND
			2006			1/67 (1.5)	0/1 (0.0)	ND
*Myonycteris torquata*	Congo	Lebango	2006	F	Y	3/91 (3.3)	0/3 (0.0)	ND
		Mbomo	2006			0/9 (0.0)	ND	ND
**Cave-dwelling bats**								
*Coleura afra*	Gabon	Batouala	2009	I	Y	5/12 (41.7)	2/3 (66.6)	1/1 (100.0)
		Faucon	2009			1/2 (50.0)	1/1 (100.0)	1/1 (100.0)
*Hipposideros cf. caffer*	Gabon	Batouala	2009	I	N	2/6 (33.3)	1/2 (50.0)	1/1 (100.0)
		Faucon	2009			1/30 (3.3)	0/1 (0.0)	ND
		Zadie	2009			0/12 (0.0)	ND	ND
*Hipposideros gigas*	Gabon	Batouala	2009	I	Y	19/33 (57.6)	0/1 (0.0)	ND
		Faucon	2009			5/56 (8.9)	1/2 (50.0)	1/1 (100.0)†
		Zadie	2009			8/40 (20.0)	0/1 (0.0)	ND
*Miniopterus inflatus*	Gabon	Batouala	2009	I	Y	9/49 (18.4)	2/4 (50.0)	1/1 (100.0)†
		Faucon	2009			0/1 (0.0)	ND	ND
		Zadie	2009			0/1 (0.0)	ND	ND
*Myotis dasycneme*	Germany	Methorst	2008	I	Y	1/25 (4.0)	ND	ND
*Myotis daubentonii*	Germany	Bavaria	2002	I	Y	0/1 (0.0)	ND	ND
		Berlin	ND			0/1 (0.0)	ND	ND
		Hannover	2003			0/1 (0.0)	ND	ND
		Hannover	ND			0/1 (0.0)	ND	ND
		Natternburg	ND			0/1 (0.0)	ND	ND
		ND	2003			0/1 (0.0)	ND	ND
		Rehburg	2003			0/1 (0.0)	ND	ND
		Wahlstorf	2009			1/18 (5.6)	ND	ND
		Wunstorf	2003			0/3 (0.0)	ND	ND
*Nyctalus noctula*	Germany	Bodenholz	2009	I	Y	0/24 (0.0)	ND	ND
*Rhinolophus spec.*	Gabon	Faucon	2009	I	N	0/1 (0.0)	ND	ND
		Mine	2009			0/15 (0.0)	ND	ND
*Rousettus aegyptiacus*	Gabon	Batouala	2009	F	Y	8/15 (53.3)	ND	ND
		Hopital	2005			1/60 (1.7)	ND	ND
		Keri	2005			4/18 (22.2)	ND	ND
		Loby-Mbigou	2005			0/9 (0.0)	ND	ND
		Massikaa	2005			0/5 (0.0)	ND	ND
		Ngounie	2005			0/1 (0.0)	ND	ND
		Zadie	2009			35/89 (39.3)	3/4 (75.0)	0/1 (0.0)†
**Total**						**114/1,135 (10.0)**	**11/30 (36.7)**	**5/7 (71.4)**†

^*^According to “Convention on the conservation of migratory species of wild animals” (www.cms.int).

^†^4/11 CCHFV IIFT and CCHF VLP reactive bat sera (1× *Hipposideros gigas*, 1× *Miniopterus inflatus*, 2× *Rousettus aegyptiacus*) were not analyzed by CCHFV NT due to sample limitation.

Abbreviations: Barro Colorado Island (BCI); not determined (ND); frugivorous (F); insectivorous (I); yes (Y); no (N).

**Table 2:** Detailed analysis of African bat sera tested in all serological assays (IIFT, CCHF VLP and NT).

No.	Species	Country	Site	Year	Roosting	Gender	Diet	Migratory*	Age	CCHF VLP NT80†	CCHFV-GP IIFT‡	CCHFV NT90§
1	*Coleura afra (GB279)*	Gabon	Faucon	2009	CD	FE	I	Y	A	Pos.	640	80
2	*Coleura afra (GB429)*	Gabon	Batouala	2009	CD	M	I	Y	A	Pos.	1,280	160
3	*Epomops franqueti (GB2497)*	Congo	Lebango	2006	FL	M	F	Y	A	Pos.	1,280	<40
4	*Hipposideros cf. caffer (GB805)*	Gabon	Batouala	2009	CD	M	I	N	A	Pos.	640	160
5	*Hipposideros gigas (GB937)*	Gabon	Faucon	2009	CD	FE	I	Y	A	Pos.	1,280	160
6	*Miniopterus inflatus (GB470)*	Gabon	Batouala	2009	CD	ND	I	Y	ND	Pos.	640	80
7	*Rousettus aegyptiacus (GB538)*	Gabon	Zadie	2009	CD	FE	F	Y	A	Pos.	160	<40

^*^According to “Convention on the conservation of migratory species of wild animals” (www.cms.int).

^†^An arbitrary reduction of 80% of *Renilla* luciferase activity was rated as positive. A serum dilution of 1:100 was applied.

^‡^Endpoint antibody titers were determined on slides containing CCHFV GP-expressing cells in a range 1:40 until 1:2,560.

^§^CCHFV serum antibody titer at 90% FFU reduction using a starting dilution of 1:40. Scarcity of bat serum samples did not allow to test lower serum dilutions.

Abbreviations: cave-dwelling (CD); foliage-living (FL); female (FE); male (M); insectivorous (I); frugivorous (F); yes (Y); no (N); Adult (A); not determined (ND).
