# Supplementary material for: Evidence for widespread infection of African bats with Crimean-Congo hemorrhagic fever-like viruses
Source: Sci Rep. 2016 May 24;6:26637. doi: 10.1038/srep26637 (PMC4877572; doi:10.1038/srep26637)
Supplement: Supplementary Information [file srep26637-s1.doc]

**APPENDIX**

**Evidence for widespread infection of African bats with Crimean-Congo hemorrhagic fever-like viruses**

**Marcel A. Müller, Stéphanie Devignot, Erik Lattwein, Victor Max Corman, Gaël D. Maganga, Florian Gloza-Rausch, Tabea Binger, Peter Vallo, Petra Emmerich, Veronika M. Cottontail, Marco Tschapka, Samuel Oppong, Jan Felix Drexler, Friedemann Weber, Eric M. Leroy, Christian Drosten**

**Supplementary Figure**

**
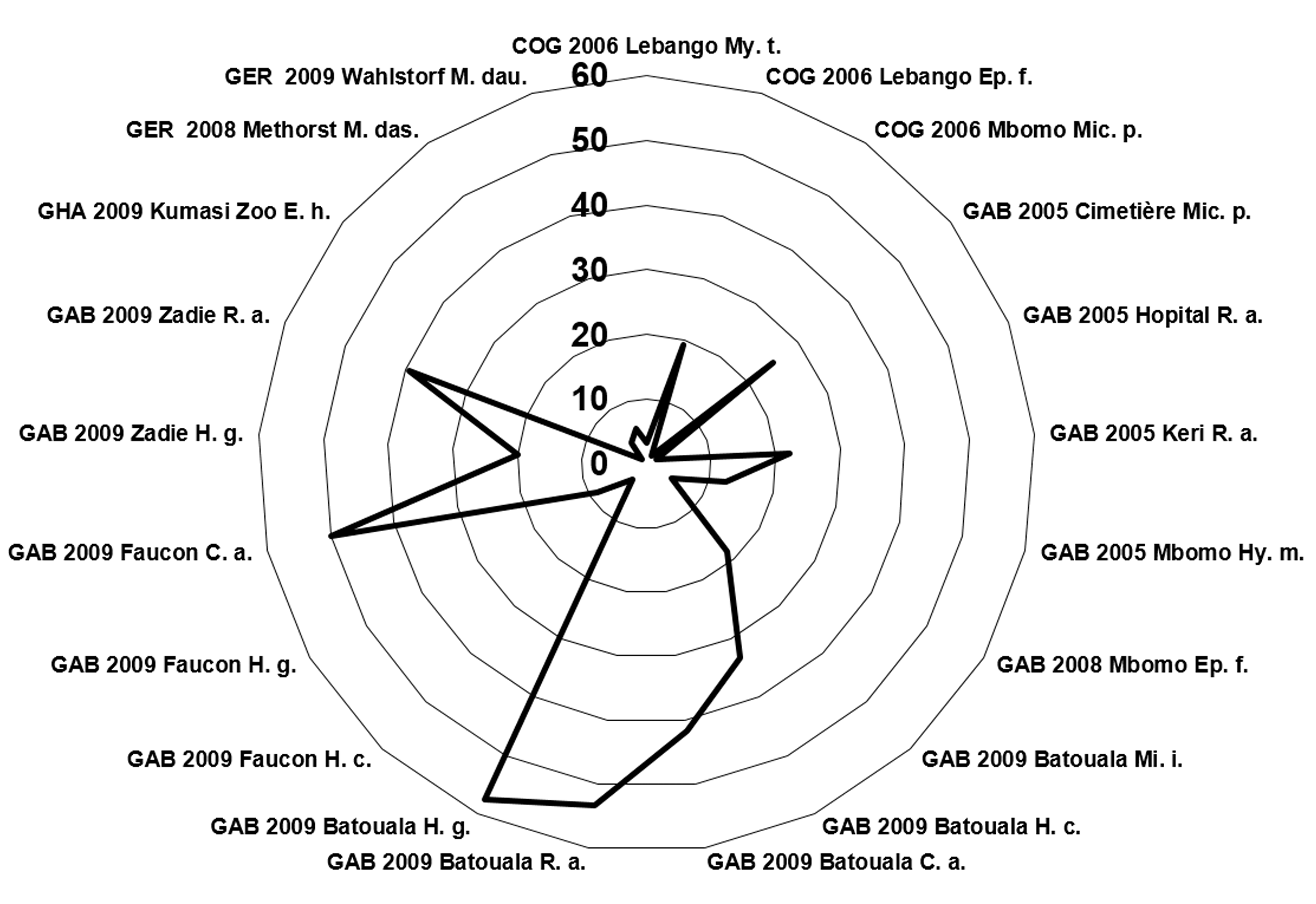
**

**Supplementary Figure.** Geographical and temporal distribution of CCHFV IIFT-positive bat serum samples. Percentage of CCHFV seropositivity was calculated per country, year, site and species. In total, 114 of 1,135 (10.0%) sera from 12 of 16 bat species sampled between 2005 and 2009 reacted with recombinant CCHFV GP antigen (range 1.0-57.6%). Highest seroprevalence was detected in bat species from the Batouala cave in Gabon, sampled in 2009 (range 18.4-57.6%). Abbreviations: COG, Congo; GAB, Gabon; GER, Germany. E. h.=*Eidolon helvum*, E. f.=*Epomops franqueti*, Hyp. m.=*Hypsignathus monstrosus,* Mic. p.=*Micropteropus pusillus*, My. t.=*Myonycteris torquata*, R. a.=*Rousettus aegyptiacus*, C. a.=*Coleura afra*, H. c.=*Hipposideros cf. caffer*, H. g.=*Hipposideros gigas*, Mi. i.=*Miniopterus inflatus*, M. das.=*Myotis dasycneme*, M. dau.=*Myotis daubentonii*.

**Supplementary Table 1. Correlations of CCHFV seropositivity with life history traits and environmental factors***

| **Feature** | **Category** | **Species (N)** | **Range** | **Mean** | **SD** | ***p-value*** |
| --- | --- | --- | --- | --- | --- | --- |
| Age | adult/subadult | 12 | 0.8-54.5 | 14.3 | 15.2 | 0.7434 |
|  | juvenile | 2 | 4.5-32.1 | 18.3 | 19.5 |  |
| Dietary | frugivorous | 6 | 0.6-24.4 | 10.9 | 9.1 | 0.4622 |
|  | insectivorous | 6 | 3.6-42.9 | 16.5 | 15.4 |  |
| Gender | female | 7 | 2.0-50.0 | 18.3 | 20.3 | 0.4613 |
|  | male | 10 | 1.0-37.5 | 12.5 | 11.4 |  |
| Migration | migratory | 11 | 0.6-42.9 | 11.9 | 13.8 | 0.4788 |
|  | resident | 2 | 2.8-6.3 | 4.5 | 2.4 |  |
| Roosting | cave | 7 | 3.6-42.9 | 17.6 | 14.4 | 0.0477 |
|  | foliage | 5 | 0.6-7.1 | 2.7 | 2.6 |  |
| Seasonality | dry | 6 | 4.5-42.9 | 22.9 | 16.6 | 0.1605 |
|  | wet | 6 | 0.7-9.1 | 3.7 | 3.1 |  |

*All statistical calculations included exclusively CCHFV-antibody positive bat species. Two-tailed t-tests were performed using the openepi website.

Abbreviations: SD, standard deviation

**Supplementary Table 2. Sample codes and detailed results of Crimean-Congo hemorrhagic fever (CCHF) and Rift valley fever (RVF) virus-like particle (VLP)-based neutralization tests using 30 CCHFV indirect immunofluorescence test (IIFT)-positive and 10 CCHFV IIFT-negative bat sera**

|  |  |  |  |  | **CCHF VLP** | | |  | **RVF VLP** | | |
| --- | --- | --- | --- | --- | --- | --- | --- | --- | --- | --- | --- |
| **No.** | **Species** | **Abbreviation** | **Code** | **CCHFV IIFT** | **Mean** | **SD** | **Rating** |  | **Mean** | **SD** | **Rating** |
| - | *Mus musculus* |  | Neg. ctrl | ND | 100.0 | 0.0 | neg |  | 100.0 | 0.0 | neg |
| + | *Mus musculus* |  | Pos. ctrl | ND | 1.7 | 1.2 | pos |  | 9.5 | 3.2 | pos |
| 1 | *Coleura afra* | C. a. | GB279 | pos | 5.1 | 3.2 | pos |  | 71.2 | 4.7 | neg |
| 2 |  |  | GB462 | pos | 45.7 | 20.6 | neg |  | 85.0 | 27.9 | neg |
| 3 |  |  | GB429 | pos | 9.3 | 3.0 | pos |  | 87.6 | 14.0 | neg |
| 4 |  |  | GB1206 | pos | 28.4 | 13.7 | neg |  | 63.3 | 44.0 | neg |
| 5 | *Epomops franqueti* | Ep. f. | GB2497 | pos | 7.8 | 4.9 | pos |  | 131.6 | 65.1 | neg |
| 6 |  |  | GB2495 | pos | 49.8 | 13.8 | neg |  | 169.9 | 84.6 | neg |
| 7 |  |  | GB3532 | pos | 81.0 | 21.4 | neg |  | 168.1 | 82.6 | neg |
| 8 |  |  | GB3517 | pos | 77.2 | 15.3 | neg |  | 118.4 | 47.3 | neg |
| 9 | *Eidolon helvum* | E. h. | GH77 | pos | 83.9 | 50.1 | neg |  | 120.8 | 35.0 | neg |
| 10 | *Hipposideros caffer* | H. c. | GB805 | pos | 9.8 | 4.2 | pos |  | 32.1 | 12.9 | neg |
| 11 |  |  | GB410 | pos | 53.1 | 14.3 | neg |  | 82.9 | 18.8 | neg |
| 12 |  |  | GB359 | pos | 31.4 | 10.0 | neg |  | 60.4 | 19.3 | neg |
| 13 | *Hipposideros gigas* | H. g. | GB498 | pos | 16.3 | 11.9 | pos |  | 73.5 | 30.1 | neg |
| 14 |  |  | GB937 | pos | 10.0 | 12.6 | pos |  | 88.7 | 15.8 | neg |
| 15 |  |  | GB682 | pos | 21.4 | 8.0 | neg |  | 97.8 | 13.8 | neg |
| 16 |  |  | GB900 | pos | 28.2 | 15.2 | neg |  | 88.6 | 18.8 | neg |
| 17 | *Hypsignathus monstrosus* | Hy. m. | GB1852 | pos | 74.0 | 43.0 | neg |  | 240.8 | 6.5 | neg |
| 18 | *Miniopterus inflatus* | Mi. i. | GB485 | pos | 31.2 | 10.1 | neg |  | 152.4 | 64.4 | neg |
| 19 |  |  | GB423 | pos | 58.3 | 21.0 | neg |  | 119.0 | 46.1 | neg |
| 20 |  |  | GB470 | pos | 1.6 | 0.8 | pos |  | 136.1 | 62.2 | neg |
| 21 |  |  | GB850 | pos | 19.0 | 10.5 | pos |  | 106.7 | 40.1 | neg |
| 22 | *Micropteropus pusillius* | Mic. p. | GB2651 | pos | 65.9 | 19.8 | neg |  | 258.9 | 9.3 | neg |
| 23 |  |  | GB1722 | pos | 40.8 | 1.8 | neg |  | 252.8 | 35.5 | neg |
| 24 | *Myonycteris torquata* | My. t. | GB2534 | pos | 86.3 | 17.8 | neg |  | 165.5 | 76.6 | neg |
| 25 |  |  | GB2535 | pos | 88.4 | 27.1 | neg |  | 274.7 | 82.8 | neg |
| 26 |  |  | GB2735 | pos | 90.4 | 7.2 | neg |  | 197.2 | 89.3 | neg |
| 27 | *Rousettus aegyptiacus* | R. a. | GB538 | pos | 14.4 | 4.1 | pos |  | 101.2 | 20.5 | neg |
| 28 |  |  | GB551 | pos | 35.0 | 3.8 | neg |  | 93.6 | 13.4 | neg |
| 29 |  |  | GB572 | pos | 12.0 | 9.9 | pos |  | 89.2 | 13.2 | neg |
| 30 |  |  | GB687 | pos | 14.2 | 11.3 | pos |  | 47.5 | 6.8 | neg |
| 31 | *Coleura afra* | C. a. | GB437 | neg | 62.6 | 24.7 | neg |  | 78.5 | 22.1 | neg |
| 32 | *Epomops franqueti* | Ep. f. | GB2496 | neg | 89.0 | 28.3 | neg |  | 208.2 | 58.3 | neg |
| 33 | *Eidolon helvum* | E. h. | GH73 | neg | 107.5 | 61.5 | neg |  | 118.0 | 32.5 | neg |
| 34 | *Hipposideros caffer* | H. c. | GB368 | neg | 45.0 | 8.8 | neg |  | 64.5 | 22.8 | neg |
| 35 | *Hipposideros gigas* | H. g. | GB1142 | neg | 38.9 | 19.8 | neg |  | 87.0 | 12.8 | neg |
| 36 | *Hypsignathus monstrosus* | Hy. m. | GB1965 | neg | 88.6 | 5.1 | neg |  | 191.3 | 72.7 | neg |
| 37 | *Miniopterus inflatus* | Mi. i. | GB851 | neg | 90.9 | 21.7 | neg |  | 138.7 | 44.1 | neg |
| 38 | *Micropteropus pusillius* | Mic. p. | GB2609 | neg | 59.2 | 10.3 | neg |  | 156.7 | 75.6 | neg |
| 39 | *Myonycteris torquata* | My. t. | GB2720 | neg | 95.3 | 40.5 | neg |  | 133.8 | 68.5 | neg |
| 40 | *Rousettus aegyptiacus* | R. a. | GB137 | neg | 73.2 | 33.5 | neg |  | 124.0 | 23.8 | neg |

Abbreviations: SD, standard deviation

**Supplementary Table 3. Detailed sampling time points and CCHFV IIFT results**

| **Species** | **Country** | **Year** | **Date sampling** | **Season** | **IIFT neg.** | **IIFT pos.** | **Total N** |
| --- | --- | --- | --- | --- | --- | --- | --- |
| *Artibeus jamaicensis* | Panama | 2011 | 01.03.2011 | dry | 17 |  | 17 |
|  |  |  | 01.04.2011 | dry | 11 |  | 11 |
| *Artibeus lituratus* | Panama | 2011 | 01.03.2011 | dry | 8 |  | 8 |
|  |  |  | 01.04.2011 | dry | 7 |  | 7 |
| *Coleura afra* | Gabon | 2009 | 30.11.2009 | dry | 1 | 1 | 2 |
|  |  |  | 01.12.2009 | dry | 7 | 4 | 11 |
|  |  |  | 10.12.2009 | dry |  | 1 | 1 |
| *Eidolon helvum* | Ghana | 2009 | 01.03.2009 | wet | 19 |  | 19 |
|  |  |  | 01.06.2009 | wet | 18 |  | 18 |
|  |  |  | 12.11.2009 | wet | 14 |  | 14 |
|  |  |  | 13.11.2009 | wet | 14 |  | 14 |
|  |  |  | 14.11.2009 | wet | 30 | 1 | 31 |
|  |  | 2010 | 12.01.2010 | dry | 10 |  | 10 |
|  |  |  | 13.01.2010 | dry | 2 |  | 2 |
|  |  |  | 08.02.2010 | dry | 17 |  | 17 |
|  |  |  | 03.03.2010 | wet | 10 |  | 10 |
|  |  |  | 10.03.2010 | wet | 11 |  | 11 |
|  |  |  | 17.03.2010 | wet | 11 |  | 11 |
|  |  |  | 31.03.2010 | wet | 4 |  | 4 |
|  |  |  | 06.04.2010 | wet | 4 |  | 4 |
|  |  |  | 13.04.2010 | wet | 2 |  | 2 |
| *Epomops franqueti* | Gabon | 2008 | 15.02.2008 | dry | 14 |  | 14 |
|  |  |  | 16.02.2008 | dry | 8 |  | 8 |
|  |  |  | 17.02.2008 | dry | 20 | 2 | 22 |
|  | Republic of Congo | 2006 | 17.05.2006 | wet | 10 | 4 | 14 |
|  |  |  | 18.05.2006 | wet | 20 |  | 20 |
|  |  |  | 19.05.2006 | wet | 9 |  | 9 |
|  |  |  | 20.05.2006 | wet | 11 | 1 | 12 |
| *Hipposideros cf. caffer* | Gabon | 2009 | 30.11.2009 | dry | 29 | 1 | 30 |
|  |  |  | 01.12.2009 | dry |  | 1 | 1 |
|  |  |  | 05.12.2009 | dry | 14 | 1 | 15 |
|  |  |  | 06.12.2009 | dry | 2 |  | 2 |
| *Hipposideros gigas* | Gabon | 2009 | 29.11.2009 | dry | 11 | 4 | 15 |
|  |  |  | 01.12.2009 | dry | 9 | 15 | 24 |
|  |  |  | 02.12.2009 | dry | 17 | 3 | 20 |
|  |  |  | 05.12.2009 | dry | 4 | 2 | 6 |
|  |  |  | 06.12.2009 | dry | 3 | 3 | 6 |
|  |  |  | 07.12.2009 | dry | 51 | 5 | 56 |
|  |  |  | 10.12.2009 | dry | 2 |  | 2 |
| *Hypsignathus monstrosus* | Gabon | 2005 | 06.04.2005 | wet | 2 |  | 2 |
|  |  | 2006 | 27.05.2006 | wet | 3 |  | 3 |
|  |  |  | 28.05.2006 | wet | 2 |  | 2 |
|  |  |  | 29.05.2006 | wet | 1 |  | 1 |
|  |  |  | 30.05.2006 | wet | 1 |  | 1 |
|  |  | 2008 | 15.02.2008 | dry | 3 |  | 3 |
|  |  |  | 16.02.2008 | dry | 2 |  | 2 |
|  |  |  | 17.02.2008 | dry | 6 |  | 6 |
|  |  |  | 23.02.2008 | dry | 2 |  | 2 |
|  | Republic of Congo | 2003 | 03.07.2003 | dry | 16 |  | 16 |
|  |  |  |  | wet | 1 |  | 1 |
|  |  | 2005 | 27.05.2005 | wet | 13 | 1 | 14 |
|  |  |  | 28.06.2005 | dry | 12 |  | 12 |
|  |  | 2006 | 17.05.2006 | wet | 4 |  | 4 |
|  |  |  | 18.05.2006 | wet | 2 |  | 2 |
|  |  |  | 19.05.2006 | wet | 2 |  | 2 |
|  |  |  | 20.05.2006 | wet | 3 |  | 3 |
|  |  |  | 21.05.2006 | wet | 2 |  | 2 |
|  |  |  | 22.05.2006 | wet | 5 |  | 5 |
|  |  |  | 23.05.2006 | wet | 2 |  | 2 |
|  |  |  | 24.05.2006 | wet | 7 |  | 7 |
|  |  |  | 25.05.2006 | wet | 2 |  | 2 |
| *Micropteropus pusillus* | Gabon | 2005 | 06.04.2005 | wet | 17 | 1 | 18 |
|  | Republic of Congo | 2005 | 27.05.2005 | wet | 13 |  | 13 |
|  |  | 2006 | 16.05.2006 | wet | 2 |  | 2 |
|  |  |  | 17.05.2006 | wet | 2 |  | 2 |
|  |  |  | 18.05.2006 | wet | 50 | 1 | 51 |
|  |  |  | 21.05.2006 | wet | 7 |  | 7 |
|  |  |  | 23.05.2006 | wet | 6 |  | 6 |
|  |  |  | 25.05.2006 | wet | 1 |  | 1 |
| *Miniopterus inflatus* | Gabon | 2009 | 01.12.2009 | dry | 26 | 7 | 33 |
|  |  |  | 02.12.2009 | dry | 1 |  | 1 |
|  |  |  | 05.12.2009 | dry | 2 |  | 2 |
|  |  |  | 06.12.2009 | dry | 10 | 2 | 12 |
|  |  |  | 07.12.2009 | dry | 1 |  | 1 |
|  |  |  | 10.12.2009 | dry | 2 |  | 2 |
| *Myonycteris torquata* | Republic of Congo | 2006 | 17.05.2006 | wet | 37 | 2 | 39 |
|  |  |  | 18.05.2006 | wet | 7 |  | 7 |
|  |  |  | 20.05.2006 | wet | 51 | 1 | 52 |
|  |  |  | 21.05.2006 | wet | 2 |  | 2 |
| *Myotis dasycneme* | Germany | 2008 | 20.05.2008 | spring | 12 |  | 12 |
|  |  |  | 24.07.2008 | summer | 12 | 1 | 13 |
| *Myotis daubentonii* | Germany | 2003 | ND | ND | 1 |  | 1 |
|  |  |  | 25.03.2003 | spring | 1 |  | 1 |
|  |  |  | 17.04.2003 | spring | 1 |  | 1 |
|  |  |  | 25.08.2003 | summer | 1 |  | 1 |
|  |  |  | 27.09.2003 | autumn | 1 |  | 1 |
|  |  |  | 18.11.2003 | winter | 1 |  | 1 |
|  |  |  | 10.04.2003 | spring | 1 |  | 1 |
|  |  | 2009 | 27.08.2009 | summer | 17 | 1 | 18 |
|  |  | ND | ND | ND | 3 |  | 3 |
| *Nyctalus noctula* | Germany | 2009 | 18.09.2009 | summer | 24 |  | 24 |
| *Rhinolophus spec.* | Gabon | 2009 | 01.12.2009 | dry | 7 |  | 7 |
|  |  |  | 02.12.2009 | dry | 8 |  | 8 |
|  |  |  | 07.12.2009 | dry | 1 |  | 1 |
| *Rousettus aegyptiacus* | Gabon | 2005 | 06.04.2005 | wet | 88 | 5 | 93 |
|  |  | 2009 | 29.11.2009 | dry | 22 | 5 | 27 |
|  |  |  | 01.12.2009 | dry | 1 | 2 | 3 |
|  |  |  | 02.12.2009 | dry | 26 | 20 | 46 |
|  |  |  | 05.12.2009 | dry | 6 | 10 | 16 |
|  |  |  | 10.12.2009 | dry | 6 | 6 | 12 |
|  |  |  |  |  | 1,021 | 114 | 1,135 |

Abbreviations: ND, not determined
